# Design of optical meta-structures with applications to beam engineering using deep learning

**DOI:** 10.1038/s41598-020-76225-9

**Published:** 2020-11-16

**Authors:** Robin Singh, Anu Agarwal, Brian W. Anthony

**Affiliations:** 1 Department of Mechanical Engineering, Massachusetts Institute of Technology, Cambridge, MA 02139 USA; 2 MIT Nano, Massachusetts Institute of Technology, Cambridge, MA 02139 USA; 3 Department of Materials Science and Engineering, Massachusetts Institute of Technology, Cambridge, MA 02139 USA; 4 Microphotonics Center, Massachusetts Institute of Technology, Cambridge, MA 02139 USA; 5 Materials Research Laboratory, Massachusetts Institute of Technology, Cambridge, MA 02139 USA; 6 Institute for Medical Engineering and Science, Massachusetts Institute of Technology, Cambridge, MA 02139 USA

**Keywords:** Engineering, Nanoscience and technology, Optics and photonics, Physics

## Abstract

Nanophotonics is a rapidly emerging field in which complex on-chip components are required to manipulate light waves. The design space of on-chip nanophotonic components, such as an optical meta surface which uses sub-wavelength meta-atoms, is often a high dimensional one. As such conventional optimization methods fail to capture the global optimum within the feasible search space. In this manuscript, we explore a Machine Learning (ML)-based method for the inverse design of the meta-optical structure. We present a data-driven approach for modeling a grating meta-structure which performs photonic beam engineering. On-chip planar photonic waveguide-based beam engineering offers the potential to efficiently manipulate photons to create excitation beams (Gaussian, focused and collimated) for lab-on-chip applications of Infrared, Raman and fluorescence spectroscopic analysis. Inverse modeling predicts meta surface design parameters based on a desired electromagnetic field outcome. Starting with the desired diffraction beam profile, we apply an inverse model to evaluate the optimal design parameters of the meta surface. Parameters such as the repetition period (in 2D axis), height and size of scatterers are calculated using a *feedforward deep neural network* (*DNN*) and *convolutional neural network* (*CNN*) architecture. A qualitative analysis of the trained neural network, working in tandem with the forward model, predicts the diffraction profile with a correlation coefficient as high as 0.996. The developed model allows us to rapidly estimate the desired design parameters, in contrast to conventional (gradient descent based or genetic optimization) time-intensive optimization approaches.

## Introduction

Nanophotonics is driving technological innovations in a variety of applications. The widespread use of nanophotonics requires the design of complex photonic microstructures that manipulate and guide light waves at the nanoscale. The design space of such microstructures is often high dimensional, where conventional methods fail to capture the global optimum in functionality^1,2^. One such example is an optical meta-surface which enables the miniaturization of complex cascades of optical elements on a plane. Metasurfaces derive their properties less from the base materials and more from their structure and orientation^3,4^. Optical metasurfaces are based on sub-wavelength structures oriented to capture and re-emit light with a defined phase, polarization, mode, and spectrum, allowing us to sculpt different light propagation patterns with unprecedented accuracy. Hence, they play a crucial role in engineering beam patterns in integrated photonic applications such as grating-based light coupling, Bragg gratings and flat-meta lens etc^3–5^.

Important in the design of an optical metasurface is the size, orientation, shape and distribution of the meta-atoms (individual scattering units on the surface)^5,6^. Various analytical and numerical approaches have been used to predict the electromagnetic response of metasurfaces^7^. Researchers have developed analytical models such as the Lewin, s-parameter based, and Generalized Effective Medium (GEM) model that approximate the spatial dispersion and finite effective refractive index of the meta-atoms through a reliable, effective model of epsilon^5–10^. However, these approaches are often time-consuming and are limited by their long-wavelength approximations, precluding their use in calculating the response of meta-atoms whose dimensions are comparable to the wavelength of light^10^. Hence, researchers adopt numerical approaches, relying on iterative waveform simulations that are based on finite-element method (FEM), finite-difference time-domain (FDTD) method and finite integration techniques (FIT)^7^. The major drawback of these approaches is that they are based on trial-and-error or empirical reasoning, making them inefficient for the design of highly non-linear devices^8^.

We consider a machine-learning-based approach, which is faster and more effective, to develop an inverse model for meta-surface design. With its enormous hidden layer capability, Deep Neural Network (DNN) based ML algorithms allow us to estimate complex and non-linear functions according to the Universal Approximation Theory^5,11^. Hence, it is possible to use these architectures to develop nanophotonic models/simulators that are otherwise time-intensive when using a conventional approach. Specifically, we define a forward model that predicts the electromagnetic response (EM) for given design parameters; and an inverse model that predicts the design/geometric parameters for a given EM response.

We consider inverse modeling of our photonic beam engineering structure that comprises a distribution of meta scatterers along the planar photonic waveguide. The ability to engineer different beam profiles such as Gaussian, focused and collimated beam enables improved efficiency in an integrated opto-fluidic sensor. It can be used to excite the analyte and observe on-chip fluorescence, or to perform IR spectroscopy^12–17^. It improves on other existing on-chip and bench-scale excitation methods in various ways. First, it helps scale up with the field of view by multiplexing a large number of excitation sources. Second, it provides compact hardware geometry to develop a scalable and low-cost solution. Third, this method can automate various biological analyses, such as screening and probing. Besides, it allows us to develop more efficient grating couplers for photonic integrated circuits^18^. One can engineer the beam profile to allow maximum power transmission through fiber couplers. Additionally, it also opens up the possibility of using edge emitter laser sources (semiconductor laser dies) as vertical light excitation (VSCEL like) devices required in LiDAR and 3D depth sensing applications^19^.

### Related work

Optimizing meta surface design has been of great interest to the photonics community in recent years. Many independent studies have demonstrated the use of DNN-based approaches to learn the relationship between device geometry and optical response in the forward model. Zhang et al. demonstrated the use of Artificial Neural Networks for RF and Microwave designs^20^. Gilliard et al. studied the dispersion relation of 2D photonic crystal designs using Multi-layer perceptron model^21^. Ferreira et al. used a hybrid EM optimization method enhanced with AL algorithm (MLP) to develop full-waveform numerical simulation and used it to predict the permittivity of metamaterials^22^. In a recent publication, Jiang et al. used simulator based training of GNN (Generative Neural Networks) for the inverse design of meta-surfaces^23^. Yao et al. worked on developing an intelligent nanophotonic structure using DNN to explore the enormous parameter space efficiently^11^. Kudyshev et al. optimized the topology on the metasurface based thermal emitter using machine learning. They used two coupled neural networks to implement GANs (Generative Adversarial Network) to perform unsupervised learning^24^. Lui et al. used DNN for the inverse optimization of nanophotonic structures and developed DNN based equivalent EM solver to design 1-D, 2-D, and 3-D dielectric metasurfaces^25,26^. Other works include meta-surface design to enhance the waveguide couplers^27,28^. Huang et al. showed out-of-plane waveguide-based holography^29^. Guo et al. showed chip integrated geometric meta surface for directional couplers^30^.

### Meta surface design and modeling

**Figure 1:**
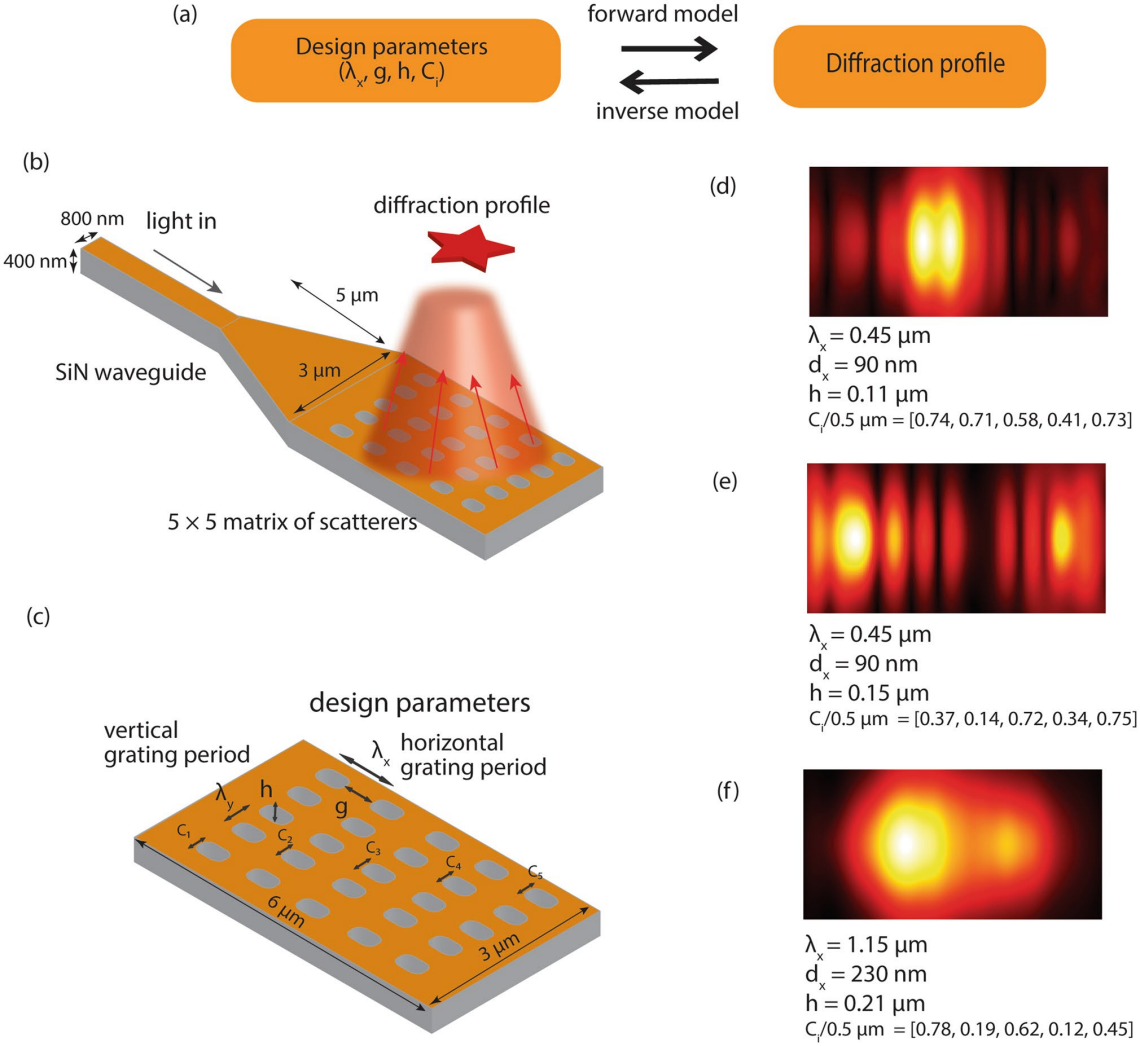
Schematic of on-chip photonic beam collimator. (**a**) Definitions of forward and inverse models defined for understanding the performance and design of metasurfaces. (**b**) SiN based photonic waveguide terminating in a meta structure that diffracts light out-of-plane. (**c**) Design parameters of the meta structure, grating period and scatterer width in horizontal and vertical directions are defined. (**d**–**f**) Top view of the diffraction profiles for different combination of the design parameters.

In this manuscript, we consider a planar meta surface composed of 5 by 5 meta scatterers distributed on the plane, which diffracts an engineered light beam in free space on top of the photonic waveguide. Figure 1b shows the photonic waveguide terminating with the meta surface fabricated in SiN material, which sits on top of a SiO_2_-On-Si wafer. We design a single-mode waveguide that supports the fundamental TE and TM modes at C and L bands of the operating wavelength. Hence, the thickness of the SiN waveguide is kept 400 nm with a width of 800 nm. The taper that combines the waveguide to the planer meta surface has a width and length of 3 μm and 5 μm, respectively.

### Theory and fundamentals

The photonic waveguides form a region with a high effective index to support orthogonally polarized fundamental modes that propagate through the grating structure. The light propagation from the structure can be understood using Huygens-Fresnel principle through constructive and destructive interference resulting from the diffraction of light from the meta surface. We assume the field distribution does not have y-direction dependence ($\frac{\partial }{{\partial y_{i} }} = 0$). Please note that the area above the meta surface is air clad. Assuming that the incoming optical wave from the left (in Fig. 1) in the generalized form $E$ = $E_{0}^{inc} (y,z)e^{i(\beta x - \omega t)}$, the diffracted light wave is given by the space harmonic field $E_{0}^{diff} (y,z)e^{{i(k_{xn} x - \omega t)}}$, where the propagation constant^31,32^

1 $$ k_{xn} = \beta_{n} + i\alpha = \beta_{0} + \frac{2n\pi }{{\lambda_{x} }} + i\alpha . $$

Here, $\beta_{n}$ is the propagation constant of the diffracted beam that depends on the periodicity of meta scatterers in the x direction, $\lambda_{x}$,$\alpha$ is the energy leakage factor and *n* refers to the diffraction order. The angle of diffraction measured from the vertical axis, $\phi_{n}$ for *n*^*th*^ order of diffraction is given by^32^

2 $$ \phi_{n} = \sin^{ - 1} \left( {\frac{{\beta_{n} }}{{k_{0} }}} \right) $$

To obtain an out-of-plane diffracted beam and avoid higher order beam diffraction, the following condition needs to be satisfied^32^

3 $$ \left| {n_{wg} - \left( {\frac{\lambda }{{\lambda_{x} }}} \right)} \right| \le \sqrt {\varepsilon_{a} } = 1, $$

4 $$ 2\left( {\frac{\lambda }{{\lambda_{x} }}} \right) - n_{wg} > \sqrt {\varepsilon_{{SiO_{2} }} } , $$

where $n_{wg}$ is the effective index of waveguide and $\varepsilon_{a}$ is the permittivity of the cladding (in this case air). Further, the leakage energy in the diffracted beam is given by ^32^

5 $$ \alpha = \alpha_{h} (\omega ,h,g)(\varepsilon_{wg} - \varepsilon_{a} )^{2} \sin^{2} (\pi \times g) $$

where $\alpha_{h} (\omega ,h,g)$ is the coefficient that depends on the light wave frequency, height and gap factor of the meta scatterers (the design parameters are defined in Fig. 1b), $\varepsilon_{wg}$ and $\varepsilon_{a}$ is the permittivity of the waveguide and air respectively.

### Design parameters

While there are many secondary parameters (e.g. the cladding thickness, substrate thickness etc.) that influence the diffraction of the light from the meta structure, we focus on the following primary parameters:

#### Periodicity (Grating Period, λ_x_)

The meta surface diffracts light waves that propagating in the waveguide in an out-of-plane direction. This results in space harmonic fields varying in the form of $E_{0}^{diff} (y,z)e^{{i(k_{xn} x - \omega t)}}$ where the propagation constant $k_{xn}$ described in Eq. (1) above depends on the grating period (λ_x_) along the propagation direction. The grating period controls the angle of diffraction as shown in Eq. (2).

#### Gap factor (g, 1 − d_x_/λ_x_)

In Eq. (5), the energy leakage $\alpha$ depends on the gap factor (g) in two ways. First, $\alpha_{h} (\omega ,h,g = 1 - \frac{{d_{x} }}{{\lambda_{x} }})$ shows slow variation of energy leakage coefficient with the gap factor. Second, gap factor critically influences the energy leakage in the form of a squared sine function where the maximum energy loss occurs when *g* = *1/2.*

#### Height (h)

Rigorous analysis described elsewhere confirms that the energy leakage depends on the etch depth of the meta scatterers (during fabrication)^32^. Etch depth controls the height of the meta scatterers (h) in the planar waveguide. For small values of the etch depth, $\alpha$ is proportional to $ED^{2}$. However, as we increase the etch depth, $\alpha$ oscillates around the saturated value of $\alpha_{T}$. The details of the dependency on the height can be found elsewhere^32^.

#### Scatterers size (C_i_)

**Figure 2:**
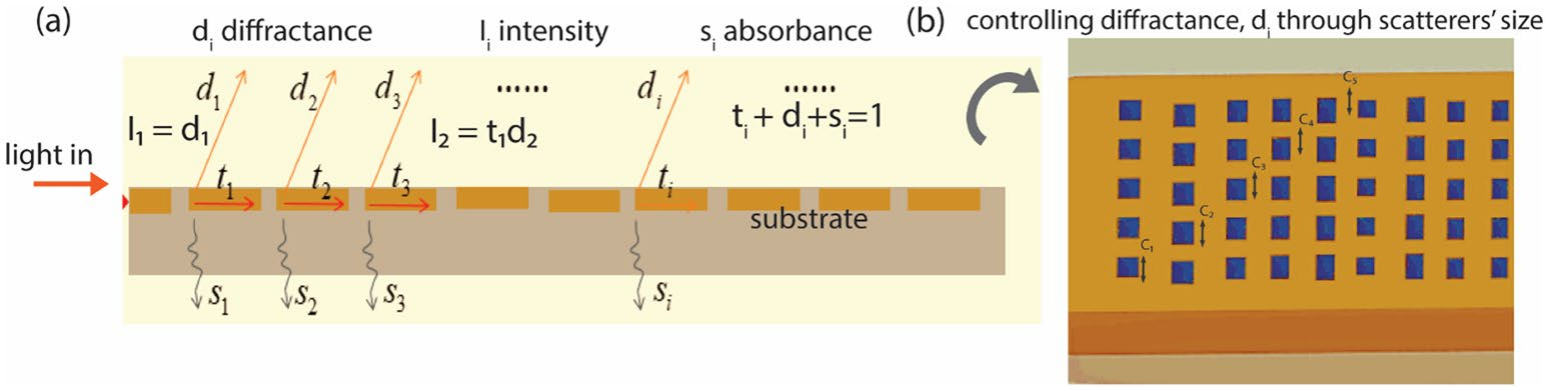
Cascade mirror model of the meta surface. Light is coupled in through a waveguide. As it propagates through individual grooves in the meta grating structure, it undergoes successive diffraction, transmission and absorption. We control the diffractance through the grooves by defining the width of the scatterers in y-direction (C_i_) as shown in (**b**).

The size of the meta scatterers controls the effective permittivity ($\varepsilon_{r}$) of the medium. This in turn influences the leakage energy of the diffracted beam. In general, the optical energy of the propagating mode tends to spread more in the denser medium (medium with higher $\varepsilon$). Hence, the size of meta scatterers in individual rows changes the leakage energy through it and controls the diffraction beam profile (as shown in Fig. 2). Specifically, we keep the width of the scatterers in the x-direction constant and only change their width in y-direction through the design parameters (C_i_) defined for each row as shown in Fig. 2b.

### Formulation of the inverse problem

Having understood the influencing factors and mechanism of the free space diffraction from the grating structure, we formulate the design problem. For the given design parameters of optical meta structure, $\lambda_{x}$, $g,ED,C_{i}$($1 \le i \le 5$), we define the forward model that computes the free space diffraction pattern. Let $\Psi (a_{1} = \lambda_{x} ,a_{2} = g = 1 - \frac{{d_{x} }}{{\lambda_{x} }},a_{3} = h,a_{4} = C_{1} ,a_{5} = C_{2} ,a_{6} = C_{3} ,a_{7} = C_{4} ,a_{8} = C_{5} )$ define the forward operator that computes the output diffraction field. Hence, $\Psi (a_{1} = \lambda_{x} ,a_{2} = g = 1 - \frac{{d_{x} }}{{\lambda_{x} }},a_{3} = h,a_{4} = C_{1} ,a_{5} = C_{2} ,a_{6} = C_{3} ,a_{7} = C_{4} ,a_{8} = C_{5} ) = I_{D} (x,y,z)$

Similarly, for the given diffraction profile, $I_{D} (x,y,z)$, we map it to the design space via $\Psi^{ - 1}$ defined as,

$$ \Psi^{ - 1} I_{D} (x,y,z) = (a_{1} = \lambda_{x} ,a_{2} = g = 1 - \frac{{d_{x} }}{{\lambda_{x} }},a_{3} = h,a_{4} = C_{1} ,a_{5} = C_{2} ,a_{6} = C_{3} ,a_{7} = C_{4} ,a_{8} = C_{5} ). $$

Hence, our defined inverse problem is to find the best estimate of $\Psi^{ - 1}$ to enable us to obtain the design parameters of the meta-grating structure.

## Results

### Feedforward Deep Neural Network (DNN)

Feedforward Deep Neural Network contains multiple hidden layers that can be used to represent an unknown complex function. The diffraction profile of the meta surface is fed into the input layer that is connected to the set of hidden layers through the activation function (ReLU). The hidden layers terminate with the output layer that predicts the design parameters of the meta surface.

### Convolutional Neural Network (CNN)

**Figure 3:**
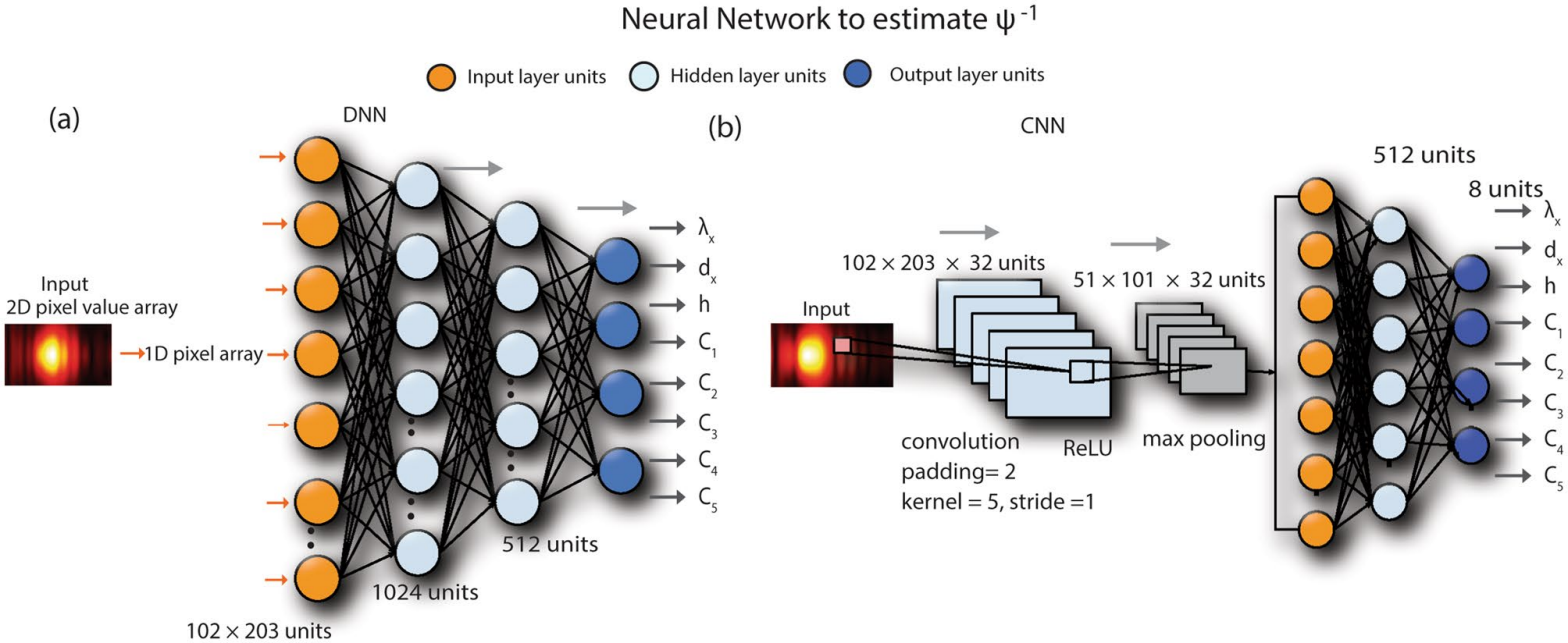
(**a**) Feedforward neural network architecture to estimate the inverse model for meta structure. Diffraction profile of the meta surface is input to the network. We use a 4-layer architecture with decreasing number of units. The output of the network is 8 design parameters of the meta structure. (**b**) Convolutional neural network (CNN) is used to report the estimation values in Fig. 5 and the text. We use a single convolution layer with 3 fully connected layers to output 8 design parameters.

In contrast to the fully connected deep neural network (DNN) with similar-sized layers, CNN has fewer connections and parameters making them easier to train. They tend to have theoretically better performance compared to the fully connected DNN with higher relative efficiency of their local architecture. Beside using fully connected DNN, we also investigate CNN architecture to estimate $\Psi^{ - 1}$ as shown in Fig. 3. It contains 1 Convolutional layer and 3 fully connected layers. The convolution filter has a padding of 2, kernel size of 5 and stride of 1. The output of the filter is passed through an activation function with non-saturating non-linearity as ReLU to improve the performance in terms of training time. Following the activation, we perform max-pooling to avoid any overfitting of the trained model. The fully connected layers follow the max pooling layer to produce 8 continuous-valued outputs.

### Performance of DNN and CNN

**Figure 4:**
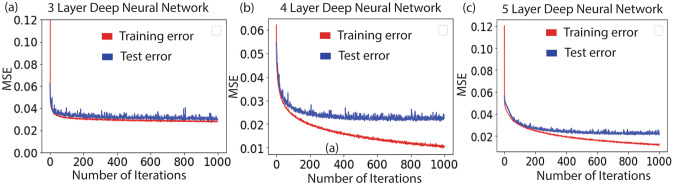
Training and test error for different architecture of the neural network. Mean square error with number of iterations for the architecture (**a**) that has 3 layer DNN. (**b**) that has 4 layer DNN (**c**) that has 5 layer DNN. The test error is minimum for the 4 layer DNN.

**Figure 5:**
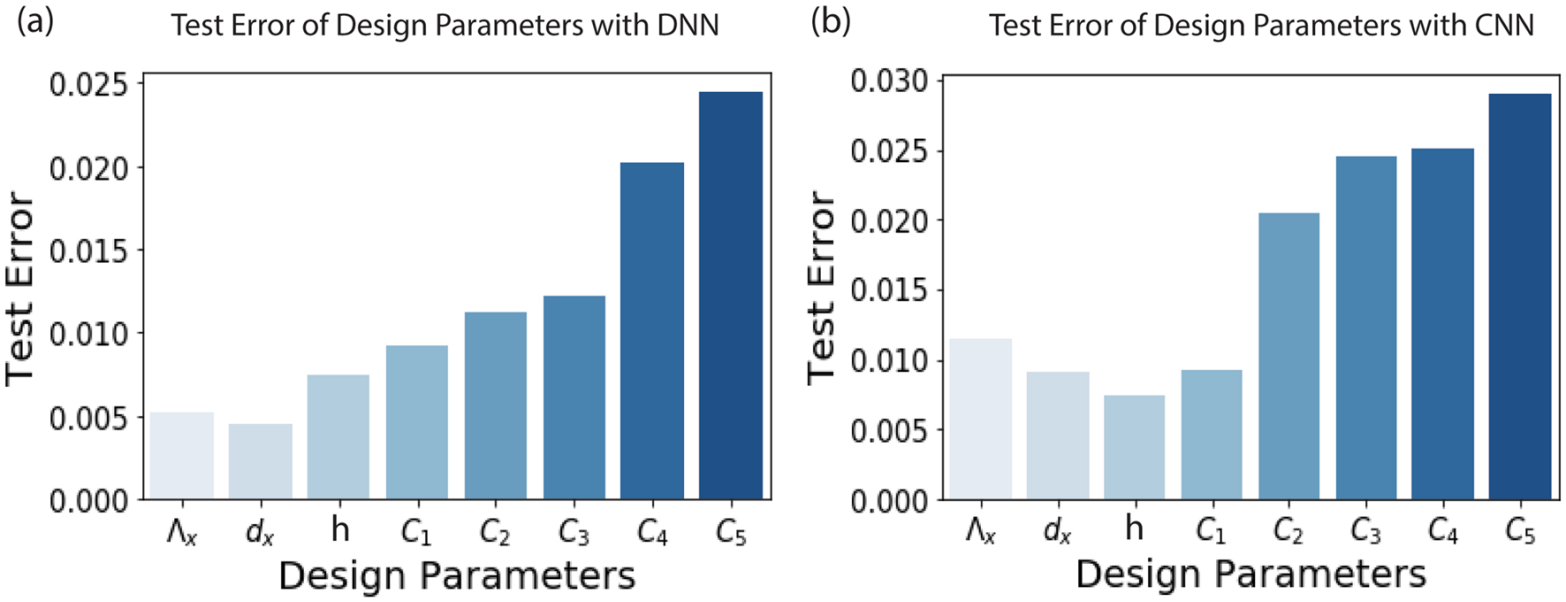
(**a**) Test error in estimating individual design parameters obtained from the best performing DNN architecture. (**b**) Test error in estimating individual design parameters obtained from the best performing CNN architecture. We conclude that DNN outperforms CNN in estimating the design parameters. The test errors in predicting $\lambda_{x}$, d_x_, h are significantly low as compared to C_i_ estimation.

We begin with the DNN consisting of 3 layers with 512 units in the hidden layer (see Fig. 4). While the training and test loss decreased with an increase in the number of iterations, there are significant jitters in the test loss. We obtain the test loss of 0.030/sample after 1000 iterations. To further investigate the effect of hidden layers in estimating the design parameters, we increase the depth of DNN to 4, consisting of 1024, 512 units in the hidden layers respectively. We obtain improvement in the predicted values with test loss of 0.012/sample after 1000 iterations. Interestingly, as we further increase the depth to 5 layers (comprising of 1024, 512 and 256 hidden units respectively), we start to see an increase in the test error. It increases to the value of 0.021/sample. We suspect the reason to be over-fitting of the data that kicks in as the number of hidden layers is increased. Figure 4 summarizes the performance of the 3 different architectures of the deep neural network. Additionally, we believe that it is possible to increase the accuracy of our model by using more training samples and by running it for more iterations. The experimentation with the neural network architecture concludes that the 4-layer deep network estimates the design parameters with the highest accuracy. Figure 5a shows the test error in estimating the individual design parameters.

We also test the performance of Convolutional neural network (CNN) and find that the training procedure is not as effective as that of DNN. We perform experiments with different CNN architectures and find that the network with single convolution layer and 3 fully connected layers gives the best estimation with test error of the design parameters as 0.0170/sample. However, due to much larger test errors in estimating the design parameters as shown in Fig. 5b, we restrict our majority of discussion to DNN for the beam engineering.

## Discussions

### Qualitative evaluations

Going further, we investigate the performance of the DNN and CNN in estimating individual design parameters. Figure 5 plots the estimation error for $\lambda_{x}$, *d*_*x*_,* h* and scatterers width (C_i_) respectively using DNN and CNN. For DNN, we observe that the error bars are relatively low for $\lambda_{x}$, *d*_*x*_,* h* (< 0.005) and C_i_ estimation is accompanied by larger inaccuracy with the error (~ 0.025). This suggests that $\lambda_{x}$, *d*_*x*_,* h* are the primary design parameters controlling the diffraction profile from the meta surface. On the other hand, higher error bands on C_i_ estimation are ascribed to ill-posed nature of the optimization problem. One can obtain the same diffraction profile with different combinations of C_i_.

**Figure 6:**
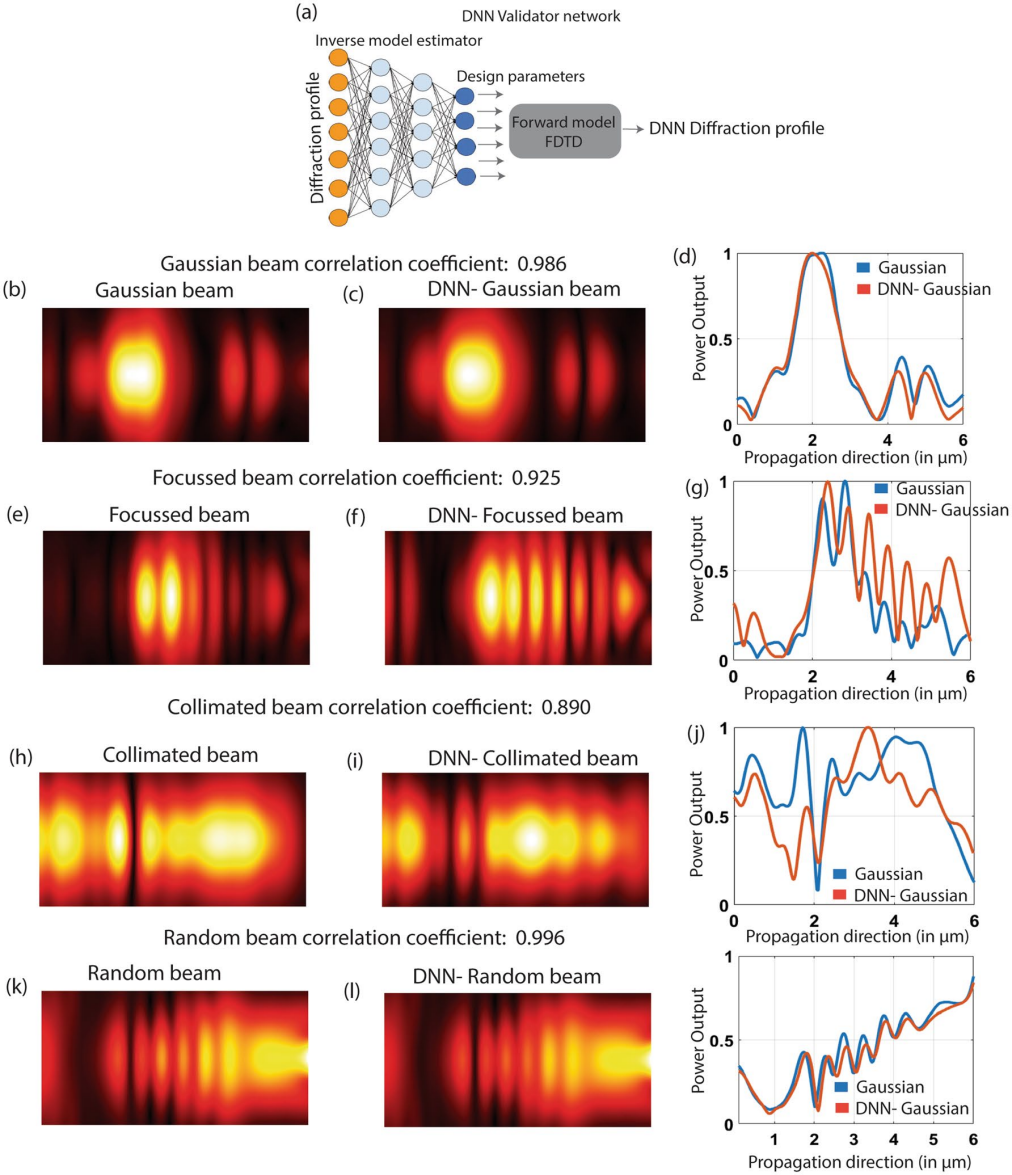
Evaluating the performance of DNN in estimating the inverse model for photonic beam engineering. The tandem DNN inverse model estimator with the FDTD forward is used to pipeline the output of DNN to FDTD simulator. (**a**) We call it the tandem network as DNN validator network. (**b**) Original Gaussian beam viewed from the top (**c**) The predicted Gaussian beam from the DNN validator network. (**d**) Comparison of the predicted and original beam profile along the propagation direction. (**e**) Original focused beam viewed from the top. (**f**) The predicted focused beam from the DNN validator network. (**g**) Comparison of the predicted and original beam profile along the propagation direction. (**h**) Original collimated beam viewed from the top (**i**) The predicted collimated beam from the DNN validator network. (**j**) Comparison of the predicted and original beam profile along the propagation direction. (**k**) Original random beam viewed from the top. (**l**) The predicted random beam from the DNN validator network. (**m**) Comparison of the predicted and original beam profile along the propagation direction.

To reason out the error analysis, we perform the forward model with the estimated design parameters. This is performed to understand the dependency of the diffraction profile on the design parameters. Figure 6 shows the diffraction profile obtained with the estimated design parameters and compares it with that of the ground truth profile. We calculate the correlation coefficient between the diffraction profile images. Mathematically, the correlation coefficient is given by^33^

$$ r = \frac{{\sum\limits_{m} {\sum\limits_{n} {(X_{mn} - \overline{X})(Y_{mn} - \overline{Y})} } }}{{\sqrt {\left( {\sum\limits_{m} {\sum\limits_{n} {(X_{mn} - \overline{X})^{2} } } } \right)\left( {\sum\limits_{m} {\sum\limits_{n} {(Y_{mn} - \overline{Y})^{2} } } } \right)} }}, $$

where $\overline{X}$ and $\overline{Y}$ are the mean of X and Y images respectively. Given that DNN outperforms CNN architecture significantly for the given training data set, we restrict our discussion to the DNN based estimator. We develop a DNN validator network based on the pre-trained inverse design model tandem pipeline (Fig. 6a). We compare different beam profiles, Gaussian, focused, collimated and random beam, estimated from the DNN with the ground truth. We find that DNN predicts the beams for Gaussian, collimated and random profile with a high correlation coefficient of 0.986, 0.925 and 0.996 respectively. However, the DNN does not perform very well in predicting the focused beam, as shown in Fig. 6, and has a correlation coefficient of 0.925. We believe that the performance of the meta surface in beam engineering can be further improved by increasing the number of scatterers in x and y directions.

### Bandwidth and power efficiency

It is ideal for the meta surface design to operate over a wide range of wavelength, particularly for cases where we intend to use them for spectroscopic applications. We calculate the 3 dB operation bandwidth of the meta structures. We find that the bandwidth is different for the different design with values of 30 nm, 24 nm, 65 nm and 70 nm for collimated, focused, Gaussian and random beam profiles respectively. Hence, we conclude that the beam profiles with extreme spatial distribution of the diffracting light tend to have smaller operating bandwidth. Further, we believe that, as we increase the degree of freedom in design parameters, for instance, apodization of grating period, the bandwidth can be improved further^34^.

## Conclusion

In the manuscript, we present the inverse modeling of optical dielectric meta-surfaces for integrated photonic applications. We develop the inverse model for a demonstration meta surface i.e., planar waveguide-based surface to engineer out-of-plane diffraction beams. We demonstrate the engineering of different beam profiles such as, uniform, focused or Gaussian.

We use a Deep Neural Network (DNN) and Convolutional Neural Network (CNN) based models to estimate the inverse operator that maps the free space diffraction profile of the metasurface to its design space composed of the grating period, gap factor, height and size of meta scatterers on the planar waveguide. We find that DNN performed better than CNN based function estimator for the given training set. While most conventional gradient descent iterative design approaches are time-consuming and computationally expensive, the developed inverse model allows us to rapidly estimate the design parameters of the meta surface, given the free space diffraction profile.

Given the generality of the approach that we implemented here, the presented machine learning-based method can easily be adapted to design complex photonic structures with high dimension design spaces. Further, our method can be used for designing an apodized meta grating structure to enhance the grating coupler efficiency as well^18^. In general, the proposed method provides a paradigm for developing and analyzing various nanophotonic structures where the conventional approach fails to yield a faithful model.

## Methods

### FDTD simulation

The electromagnetic simulation of optical meta-surfaces is performed using the finite difference time domain (FDTD) simulation implemented using commercially Lumerical software (Lumerical Inc., Vancouver, BC, Canada). We consider a photonic planar 400 nm thick SiN based waveguide structure on 3 µm SiO_2_ substrate with air cladding on top of the waveguide. The refractive index of SiN and SiO2 are taken as 1.91 and 1.414, respectively at λ = 1550 nm. In the manuscript, we use FDTD for two major purposes; to develop exhaustive training data set and to achieve the DNN validator network based on the pre-trained inverse design model tandem pipeline.

### Data generation, formatting and preprocessing

We use the random parameter combination of grating period ($\lambda_{x}$), scatterer size in x direction ($d_{x}$) scatterer size in y direction for each row $C_{i}$ (0 < *i* < 6) and height (h) of the meta surface in python script and combined it with Python API of Lumerical Software. The API integrates the Lumerical tools with Python enabling us to automate the data generation. Lumerical FDTD software performs the full wave 3D FDTD simulation. The beam diffraction profile of the grating structure (viewed from the top) is stored as an RGBP image labeled with its corresponding design parameter values. Specifically, we vary ‘λ_x_’ between 0.3 to 1.4 µm, ‘$d_{x}$’ in range between 50 nm to 1.2 µm, ‘h’ between 100 to 400 nm and ‘$C_{i}$’ in ranges between 50 to 480 nm. Different combinations of these design parameters are used to generate the diffraction beam profiles. We generate about 4000 training dataset with these design parameters. The input light wavelength is kept constant at 1.5 µm, most commonly used wavelength range in the integrated photonic applications.

### Network generation

#### Training procedure

Neural Network is implemented through Pytorch library on Python. As such, we begin with defining the neural network architecture using the hidden layers and units. We test with different architectures to optimize the estimation error. The output from the hidden units is passed through the activation function. It is followed by defining the loss function and optimizer to estimate the values of the parameters. Once the appropriate functions are defined in the software, we perform gradient decent based optimization to estimate the model parameters.

#### Hyperparameters, loss and activation function

Our DNN based machine learning algorithms require an optimal selection of hyperparameters such as batch size, number of iterations and learning rate. Batch size is one of the most important hyper parameters to tune in DNN models. On the one extreme, larger batch size guarantees convergence to the global optimum of the objective function, on the other extreme, smaller batch size has a faster convergence rate to a "good" solution, but does not guarantee a global optimum of the objective function. We choose a batch size of 125 for training dataset of about 4000 images to feed in the images to the training network. Further, we use stochastic gradient descent (SGD) algorithm to find the local/global maxima/minima in our design space. SGD typically requires an ideal learning rate and number of iterations to explore the design space and reach the local extremum of the optimization function. For our training, we use learning rate of 0.06 and 1000 iterations. We find that increasing the learning rate resulted in training jitters and increased training loss. Similarly, after 1000 iteration, the change in the training and test loss are insignificant.

Among other aspects, we need to choose the loss and activation function. Given the values to be predicted are continuous real positive values, we use ReLU as the activation function for all the hidden layers. Further, it helps to improve the convergence rate of the model as well. As a part of the optimization algorithm, the error for the current state of the neural network model must be estimated repeatedly. This requires the choice of an error function, conventionally called a loss function that could quantify the difference between the computed output of the network and the true value. Since our neural network is designed to estimate the continuous values of the design parameters, we chose the mean square loss function. Mathematically, it is defined as

$$ MSE = \frac{1}{n}\sum\limits_{i = 1}^{n} {(Y_{i} - \hat{Y}_{i} } )^{2} . $$

## Data Availability

Lumerical script files and any other accompanied codes used for modeling and training of the meta-surface are available from the corresponding authors upon reasonable request.
